# Evaluating the echogenicity of ethyl cellulose-ethanol gel for tracking biodistribution during liver ablation

**DOI:** 10.1038/s41598-025-11336-9

**Published:** 2025-07-15

**Authors:** Jeffrey Yang, Xihan Ma, Andrew S. Mikhail, William F. Pritchard, Bradford J. Wood, Haichong K. Zhang, Jenna L. Mueller

**Affiliations:** 1 Fischell Department of Bioengineering, University of Maryland, College Park, MD USA; 2 Center for Interventional Oncology, Radiology and Imaging Sciences, Clinical Center, National Institutes of Health, Bethesda, MD USA; 3 Department of Robotics Engineering, Worcester Polytechnic Institute, Worcester, MA USA; 4 Marlene and Stewart Greenebaum Cancer Center, University of Maryland School of Medicine, Baltimore, MD USA; 5 3102 A. James Clark Hall, University of Maryland, 8278 Paint Branch Drive, College Park, MD USA 20742

**Keywords:** Ethanol ablation, Ethyl cellulose, Liver ablation, Ultrasound imaging, Ultrasound image segmentation, Medical imaging, Cancer therapy, Cancer therapy, Biomedical engineering

## Abstract

Hepatocellular carcinoma (HCC) is the third most common cause of cancer deaths worldwide. While surgery and liver transplantation are curative treatments for HCC, many tumors are unresectable due to co-morbidities or advanced stage. Ethanol ablation is an established alternative ablative therapy for HCC that is typically paired with ultrasound imaging to visualize tumors and enable precise ethanol delivery. However, ethanol has the propensity to leak from the injection site and is not inherently echogenic, making biodistribution difficult to monitor. To address these limitations, we added ethyl cellulose (EC) with ethanol to form a gel that improves ethanol retention and generates an echogenic depot in tissue. We performed studies in tissue phantoms and liver tissue to characterize the acoustic profile of the EC-ethanol depots. Studies in phantoms showed that the EC-ethanol depots were 1.5x more echogenic when EC-ethanol ratios increased from 6 to 12% (*p* < 0.001). EC-ethanol depots in excised liver tissue and in swine liver post-mortem were acoustically discernable and generated 4 cm^2^ depots, which are of clinically relevant size for HCC treatment. In summary, this study established the echogenic properties of EC-ethanol for spatiotemporal analysis of injectate distribution, demonstrating its translational potential for tracking biodistribution during liver ablation.

**Supplementary Information:**

The online version contains supplementary material available at 10.1038/s41598-025-11336-9.

## Introduction

Liver cancer is the sixth most diagnosed cancer and third highest cause of cancer deaths worldwide^1^, with hepatocellular carcinoma (HCC) accounting for approximately 80% of all primary liver cancer cases^1^. While surgical resection is a curative approach, only a minority of patients are eligible for surgery depending on tumor multifocality and adequate remnant liver volume^2^. Ablative therapies, such as radiofrequency and microwave ablation, offer effective minimally invasive technologies for localized treatment of HCC^3,4^. These treatment modalities have become standard options outside of surgery for treating liver tumors^4^. The expensive equipment and high cost of performing these procedures, however, hinders their applicability in clinics situated in low- and middle-income countries (LMICs)^4^, highlighting the critical need for cost-effective technologies to treat HCC in these settings.

Ethanol ablation is a low-cost, time-tested percutaneous treatment without costly instrumentation that has been shown to safely treat HCC tumors up to 3 cm in diameter^5^. The therapy eliminates the need for sterilization equipment, due to the use of disposable consumables. Moreover, ethanol is a relatively common chemical available in LMICs, making ethanol ablation an accessible treatment option for HCC in these regions^4^. The treatment is typically coupled with ultrasound image guidance to enable precise delivery of ethanol to tumors. While other imaging modalities, such as computed tomography (CT) and magnetic resonance imaging (MRI), offer image guidance for percutaneous treatments, ultrasound is the most accessible technology for LMICs, due to its relatively low cost, simpler maintenance, and portability^6^.

Drawbacks of ethanol ablation include (1) the tendency for ethanol to leak from the injection site into adjacent tissue structures, resulting in adverse effects and diminished treatment efficacy^7^, and (2) ethanol is not readily visible under ultrasound after injection, making biodistribution difficult to monitor^8^. To mitigate leakage, we previously combined ethanol with a polymer, ethyl cellulose (EC), which forms a solid gel once injected into tumors^9^ and helps retain ethanol at the injection site. EC-ethanol is comparable in cytotoxicity with pure ethanol and was tested in numerous preclinical tumor models, including oral squamous cell carcinoma^9^, breast cancer^10,11^, liver cancer^12,13^, and cervical pre-cancer^14–16^. All studies demonstrated improved tissue coverage^13–16^, superior stunted tumor growth, and enhanced ethanol treatment efficacy compared to pure ethanol control treatments^10–12^.

While the addition of EC improved ethanol retention in tissues, it is unclear whether the local biodistribution of EC-ethanol can be tracked and characterized via ultrasound, steps that are integral to improving US procedural guidance, enabling confirmation of on-target delivery, and ultimately, translating this technology to treat patients with HCC worldwide. This study seeks to investigate the echogenic properties of the EC-ethanol gel and evaluate its ultrasound visibility in tissue.

## Materials and methods

*Preparation of EC-ethanol solution.* Mixtures of EC (Sigma Aldrich, St. Louis, MO) and ethanol (200 proof, Koptec, King of Prussia, PA) were prepared by blending EC into the ethanol via stirring at room temperature, as previously described^15^. The ratio of EC to ethanol ranged between 6 and 12% (EC: ethanol, w:w).

*Preparation of agar phantoms.* Agar-based phantoms were used to investigate the echogenic properties of EC-ethanol in vitro. 1% agar (UltraPure Agarose, Invitrogen, Carlsbad, CA) was dissolved in degassed, deionized water (agar: water, %w: v) and heated until a clear, molten solution was obtained. The solution was then poured into a custom-made polyvinyl alcohol reservoir (3D Herndon, Herndon, VA)^17^ and cooled in 4 °C to solidify into a solid block. 10 × 10 mm standard spectrophotometer cuvettes (VWR, Radnor, PA) were placed in the middle of the agar solution within the block while cooling and then removed to form reservoirs.

**Fig. 1:**
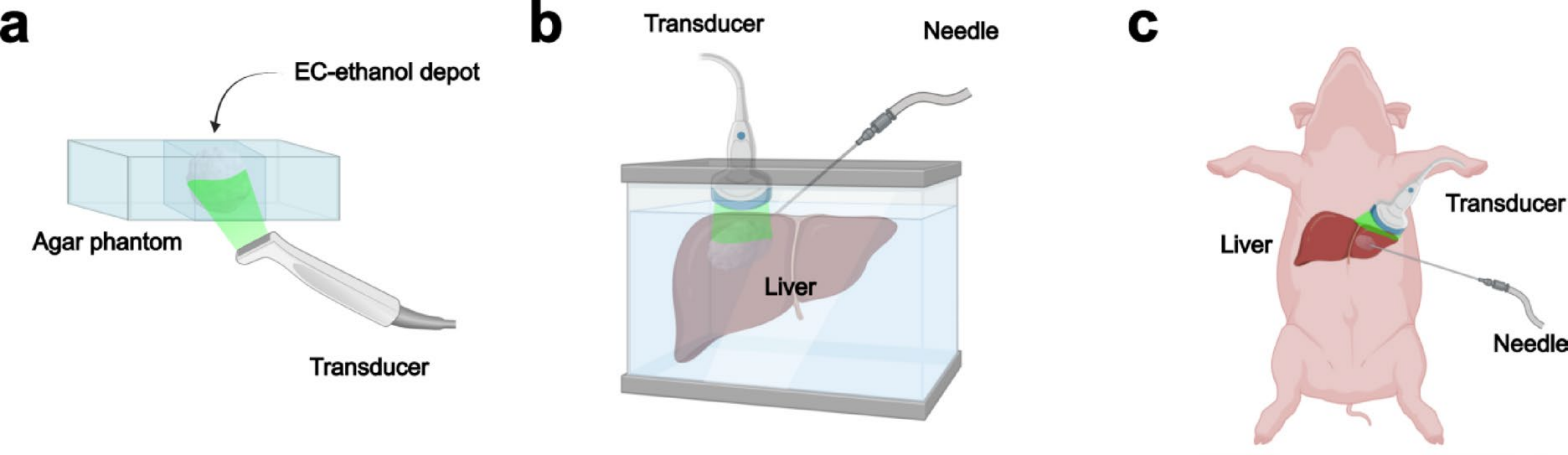
Experimental workflow schematics for evaluating the echogenic properties of EC-ethanol (**a**) in vitro, (**b**) in ex vivo bovine liver, and (**c**) in post-mortem in situ swine liver. This figure was created with Biorender.com.

*Acquisition of EC-ethanol ultrasound images.* Figure 1a illustrates the experimental setup for obtaining ultrasound images of the EC-ethanol depots in agar phantoms and examining the depot’s echogenic properties. A saline solution was first added into the reservoirs of the agar block. 1 mL of EC-ethanol was then added to the spaces and manually stirred to form a visible gel depot. An intraoperative linear array ultrasound transducer (SLAx, 6–13 MHz frequency, Sonosite M-Turbo, Fujifilm, Minato City, Tokyo, Japan) was then immediately placed on the side of the agar block to obtain B-mode images of the depot at 1 cm imaging depth. A rubber insulator was placed on the other side of the block to act as an ultrasonic attenuator to reduce the ultrasound reflections from the agar-air interface. The proximal field was defined as the region between the transducer and the front face of the EC-ethanol depot, while the distal field was defined as the region between the back end of the EC-ethanol depot and rubber insulator.

For tissue experiments, fresh bovine livers were obtained from the local butchery (Balducci’s Food Lover’s Market, Bethesda, MD). Figure 1b illustrates the experimental setup for performing injections of EC-ethanol into the livers. The liver was placed in a container with 1X PBS heated to 37°C. A curved array ultrasound transducer (C9-2, 2–9 MHz frequency, Philips Ultrasound, Bothell, WA) connected to a clinical ultrasound instrument (EPIQ 7, Philips) was fixed in place while in contact with the top surface of the liver. A rubber insulator was placed underneath the liver to serve as an ultrasonic attenuator. Syringes, 12 mL, and 18-gauge 10-cm length Chiba biopsy needles, connected with 33” extension tubing (MX451SL, Smiths Medical, San Clemente, CA), were used to inject EC-ethanol. A programmable syringe pump (PHD ULTRA™, Harvard Apparatus, Holliston, MA) was used to control the infusion volume (4 mL) and rate (100 mL/hr), as previously established^17^. A custom-made needle guide^17^ was used to insert the needle at a 45° angle along the same plane as the transducer. Once the needle and transducer were properly positioned, B-mode images of the injection procedure and resulting depot were obtained at 2 cm imaging depth. Specifically, the following B-mode images were captured in time series: tissue background with needle inserted and primed; EC-ethanol injection after 30, 60, 90, and 120 s; and EC-ethanol depot in the liver after completion of the injection.

For the in situ study, one adult male swine intact carcass (60 kg; Oak Hill Genetics, Ewing, IL) was obtained from an unrelated study approved by the National Institutes of Health Clinical Center’s Institutional Animal Care and Use Committee (DRD23-01, approved 18 Apr 2023). This study is performed in accordance with relevant guidelines and regulations. All methods are reported in accordance with ARRIVE guidelines. Swine serve as a large animal model with organs of similar size and geometry to humans, suitable for assessing the safety and efficacy of interventional devices and ablative therapies^18,19^ intended for clinical translation. Figure 1c illustrates the experimental setup for performing percutaneous injections of EC-ethanol in swine liver. A small incision in the skin was made to insert an 18-gauge 10-cm length Chiba biopsy needle and percutaneously access the liver and inject EC-ethanol under ultrasound guidance at 8 cm percutaneous imaging depth (EPIQ 7). B-mode images of the EC-ethanol depot and tissue background throughout the injection were acquired and analyzed through the same design as that detailed in the ex vivo bovine liver experiment.

**Fig. 2:**
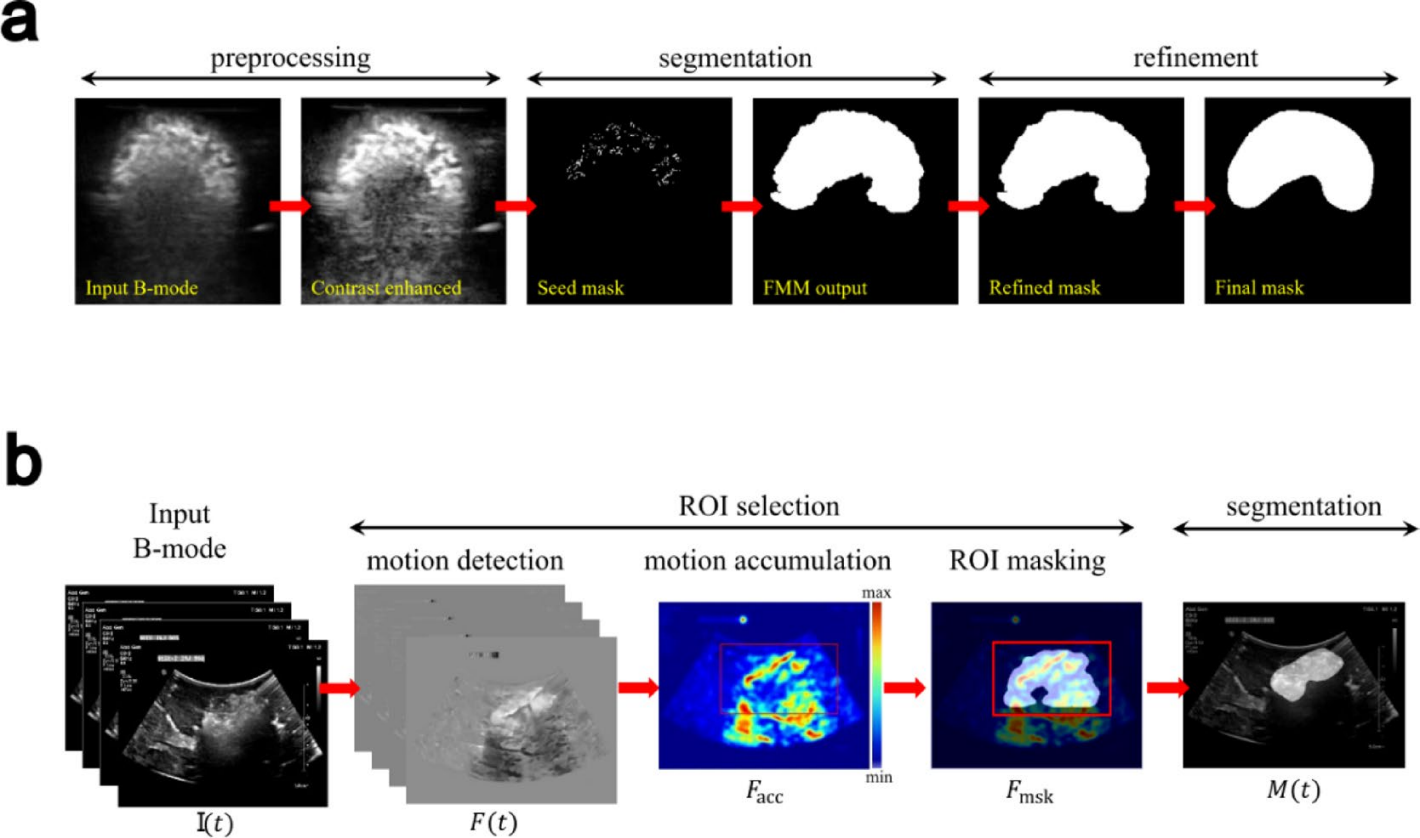
Process workflow for detecting and segmenting the EC-ethanol depot in ultrasound images for quantitative analysis. (**a**) Ultrasound image analysis of EC-ethanol depots in in vitro agar phantoms, where the depot echogenic signal in the B-mode image was processed and segmented to generate its corresponding binary mask. (**b**) Ultrasound image analysis of EC-ethanol depots in liver tissue, where motion frames, F(t), from an input time-series of the depot formation, I(t), were captured to define the ROI (red boxes) for each frame and then generate corresponding binary masks, M(t).

*Analysis of EC-ethanol ultrasound images in agar phantoms.* The depot area in B-mode images was segmented via binary masking to enable quantitative analysis. The automated mask generation process involved several key steps which are illustrated in Fig. 2a. The image processing methods were implemented using MATLAB 2023b and deployed on a workstation with Intel i9-12900 CPU and 64 GB RAM. The MATLAB script reads ultrasound images recorded from a scanner. The speed for the depot segmentation pipeline was approximately 21 frames per second. First, a rectangular region-of-interest (ROI) box was defined to encompass the depot area within the images. The B-mode images were then processed for contrast enhancement and speckle denoising within the ROI. The contrast enhancement was performed using the Contrast Limited Adaptive Histogram Equalization algorithm^20^ with a tile size equal to 2% of the input image size. The denoising was done using the non-local means filter algorithm^21^ (Supplementary Fig. S1). These steps improved the visibility of the depot area, which was characterized by higher pixel intensity compared to its surroundings. Subsequently, a preliminary mask referred to as the seed mask was generated based on intensity thresholding applied to the contrast enhanced ROI. The intensity threshold, $\:{\tau\:}_{\text{s}\text{e}\text{e}\text{d}}$, was determined by the following formula, $\:{\tau\:}_{\text{s}\text{e}\text{e}\text{d}}={\mu\:}_{\text{r}\text{o}\text{i}}+3{\sigma\:}_{\text{r}\text{o}\text{i}}$, where $\:{\mu\:}_{\text{r}\text{o}\text{i}}$ and $\:{\sigma\:}_{\text{r}\text{o}\text{i}}$ are the mean and the standard deviation of the pixel intensities within the ROI (i.e., the approximate depot area), respectively. The seed points within the depot area (i.e., bright pixels in the seed mask) were then expanded using the Fast Marching Method (FMM)^22^. FMM facilitated the propagation of the mask boundaries from the initial seed points until the fronts reached locations with sharp intensity transitions in the B-mode image, forming a mask that readily covered the region of the depot. Next, the FMM output underwent several refinement steps to eliminate any impulse noise and merge multiple islands into one whole binary mask. This involved a two-time area-opening operation which removed islands that have fewer than $\:n$ pixels, and a 2-dimensional Gaussian filtering in between. Suppose there exists $\:m$ islands after the FMM operation whose areas (i.e., number of pixels) are sorted in descending order. The first area opening keeps only the first 75th percentile of the largest islands, effectively removing impulse noises. The remaining islands located around the depot center were connected by the Gaussian filtering. The second area-opening then only keeps the largest area island, representing the depot area.

The perimeter of the visible depots was manually traced in ImageJ software (Fiji, National Institutes of Health, Bethesda, MD) before generating binary masks to serve as the ground truth when calculating the Dice Similarity Coefficient between the manually segmented depots and the aforementioned automatically segmented depots. This analysis was done to validate the automatic segmentation algorithm. Histograms of the non-zero-pixel intensities within the automatically generated ROIs were plotted for different EC-ethanol ratios to characterize echogenic signatures. Various parameters including the total depot area, perimeter of the depot, and circularity of the depot were quantified from the automatic segmented binary masks in ImageJ.

*Analysis of EC-ethanol ultrasound images in liver tissue.* Automatic depot segmentation was performed on the ex vivo and in situ liver images to assess the acoustic characteristics of the EC-ethanol depot, as described in Fig. 2b. The one-step motion map generation took 0.3 s. Taking into consideration the B-mode frames captured in time series, two assumptions were made to allow temporal tracking of the depot: (1) the depot area was expected to contain the most significant motion in the cine, stemming from volume increase during injection; (2) the size of the depot continued to increase. Assumption 1 enabled automatic ROI detection which was critical to ensure high specificity of the segmentation. The ROI detection began by calculating motion frames, F(t), for each B-mode frame captured from 0 to 120 s (t represents the timestamp):

1 $$F\left( t \right) = {\text{norm}}\left( {\sum\limits_{t = 0s}^{120s} I \left( t \right)} \right) - F{\text{ref}}$$

where I(t) is the B-mode image at time t, norm(∙) denotes the min-max normalization operation, and F_ref is a reference frame which contains only the tissue background and the needle. The resulting motion frames were then summed to create a cumulative motion map, F_acc. Higher values in F_acc indicated greater temporal motion. The region exhibiting the most significant motion was then segmented using the same pipeline introduced in Fig. 2a. The final depot ROI was defined by a rectangular region encapsulating the motion mask, with F(t) as the input instead of the B-mode image. This ROI selection algorithm was deployed prior to the depot segmentation to ensure accurate masking of the depot area within the liver tissue. Lastly, the output from the segmentation, denoted as M(t), went through temporal aggregation to fulfill assumption 2:

2 $$M(t) = M(t) \bigodot M(t-1)$$

where ⨀ is the logical OR operator. This step ensured the growth of the depot is temporally smooth. Manual segmentation of the depots was performed similarly to those in the agar phantoms to generate ground truth binary masks and corresponding Dice scores to validate the automatic segmentation method. The masks from the automatic segmentation method were measured with ImageJ to obtain the following quantitative parameters: area, perimeter, circularity. Histograms of the non-zero-pixel intensities within the ROI were plotted for different EC-ethanol ratios to characterize echogenic signatures.

*Statistical analysis.* Statistical analyses were performed using Graphpad Prism (GraphPad Software, San Diego, CA, USA). The Kruskal-Wallis non-parametric analysis of variance was used to compare the parameters obtained from EC-ethanol depots of varying EC concentrations in the in vitro and liver experiments. The Dunn’s non-parametric multiple comparisons test was then applied to identify the groups displaying significant differences. A significance level of *p* = 0.05 was applied to reject the null hypothesis in all analyses.

## Results

**Fig. 3:**
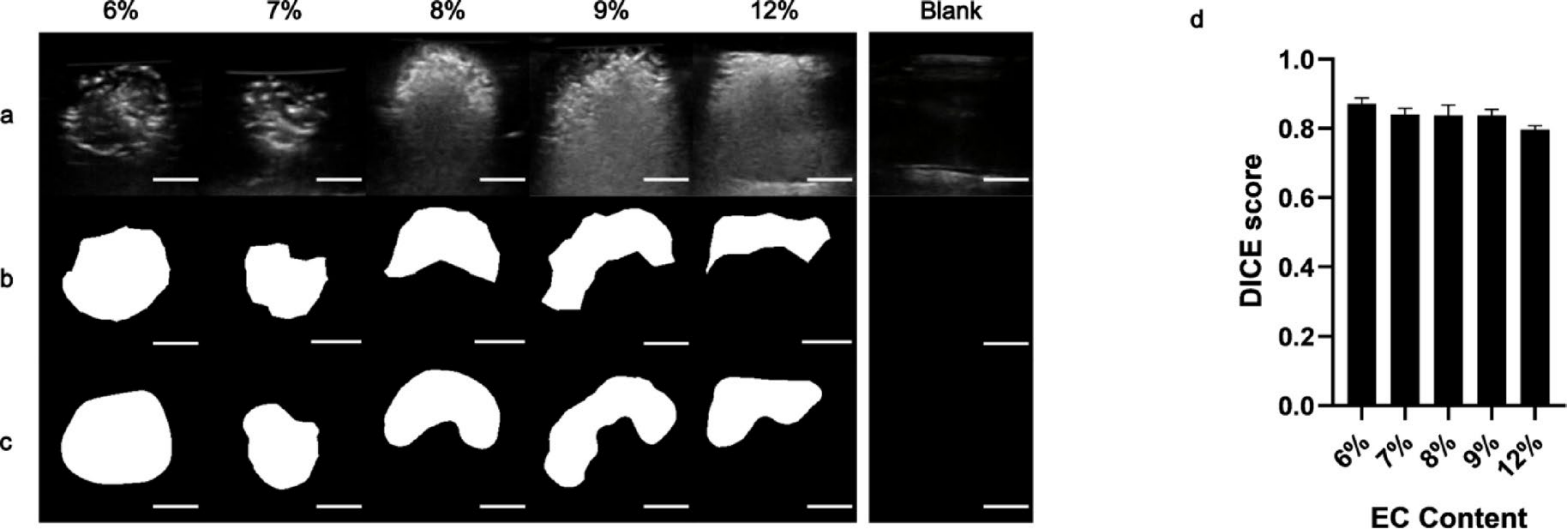
Visibility of different concentrations of EC-ethanol in agar under ultrasound imaging. (**a**) Representative B-mode images and binary masks of the EC-ethanol depots generated via (**b**) manual segmentation and (**c**) automatic segmentation. Scale bars = 5 mm. (**d**) Dice scores for measuring the similarity between binary masks generated from manual depot segmentation vs. automatic segmentation. *N* = 10.

**Fig. 4:**
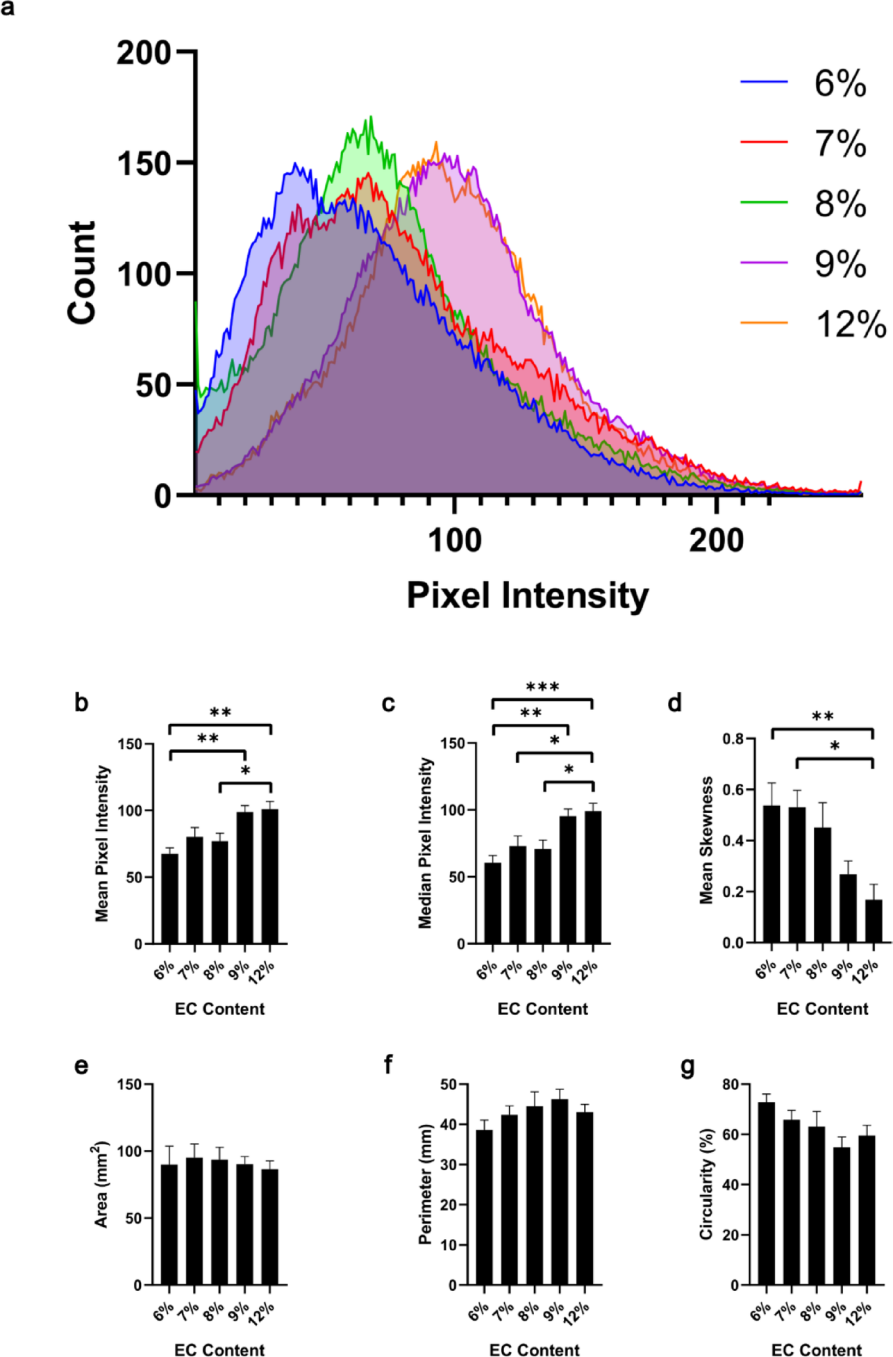
Characterization of the echogenic profile of the EC-ethanol depots in agar phantoms. (**a**) Histogram plot showing the non-zero cumulative pixel intensities observed in the EC-ethanol depot. Plots depicting the (**b**) mean, (**c**) median, and (**d**) skewness of the pixel intensity distribution. Additional plots showing the (**e**) area, (**f**) perimeter, and (**g**) circularity calculated from the binary masks. All error bars in this figure are SEM. *N* = 10. Kruskal-Wallis non-parametric analysis of variance test was performed. *P < 0.05; **P < 0.01; ***P < 0.001.

*EC-ethanol echogenicity correlates with EC content.* Representative B-mode images and binary masks of the echogenic profile of EC-ethanol depots in the agar phantoms are shown in Fig. 3. The masks corresponded to the ROI where the EC-ethanol was deposited in the agar (Fig. 3a-c), excluding any hyperechoic artifacts induced by the EC-ethanol depots. The Dice scores largely remained consistent, ranging between 0.87 ± 0.02 (6% EC) and a slightly lower score of 0.80 ± 0.01 (12% EC) (*p* > 0.05) (Fig. 3d). The pixel intensities within the ROI of the B-mode images showed the distribution of the echogenic signal varied with EC concentration; in particular, 9% and 12% EC-ethanol were shifted to the right compared to the lower EC-ethanol ratios (Fig. 4a). Both the mean and median pixel intensities of the EC-ethanol depot significantly increased as EC content increased (Fig. 4b, c) from a mean of 68 ± 4 (6% EC) to 101 ± 6 (12% EC) and a median of 60 ± 6 (6% EC) to 99 ± 6 (12% EC) (mean ± S.E.M., *n* = 10; *p* = 0.0003 and 0.0002 for mean and median, respectively). The pixel intensities of all EC-ethanol depots displayed positive skewness (Fig. 4d), though there was a significant shift toward a normal distribution as EC content increased from 6% (0.54 ± 0.09) to 12% (0.17 ± 0.06) (*p* = 0.0023). For the binary mask analysis, the average area (between 87 ± 6 and 95 ± 10 mm^2^ and perimeter (between 39 ± 2 and 46 ± 3 mm) of EC-ethanol depots between 6% and 12%, respectively, were not significantly different (*p* > 0.05) (Fig. 4e, f). Conversely, the circularity of the depots decreased as EC content increased from 0.73 ± 0.03 (6% EC) to 0.59 ± 0.04 (12% EC) (*p* = 0.063) (Fig. 4g). Due to similar echogenic properties between 9% and 12% and the difficulty of injecting high viscosity solutions^12,15^, subsequent experiments only focused on EC-ethanol solutions between 6% and 9%.

**Fig. 5:**
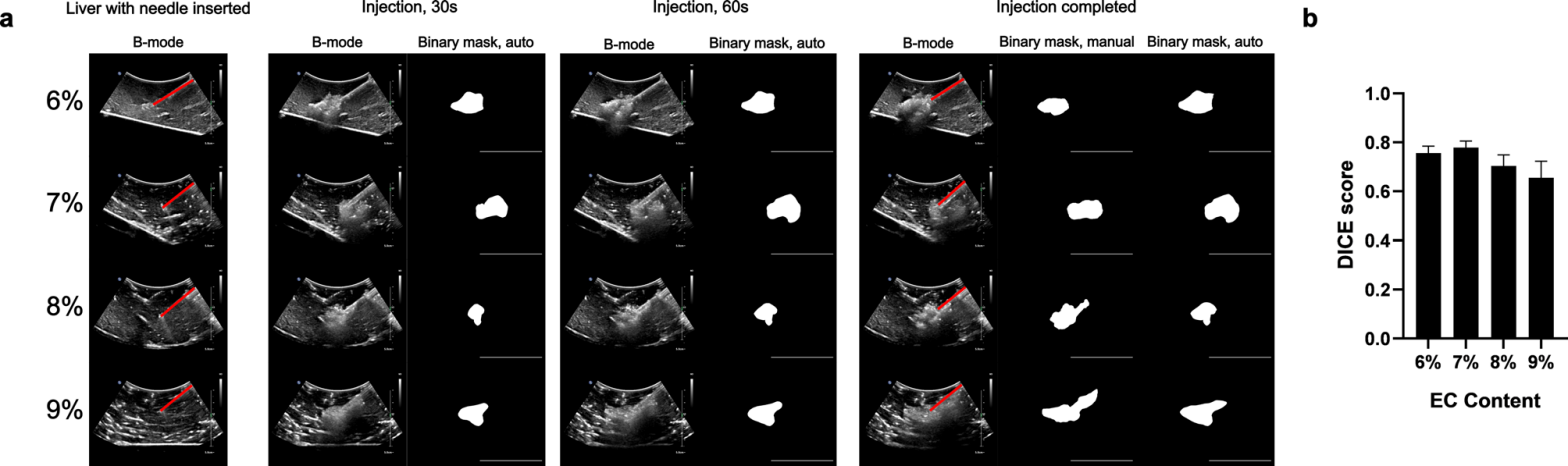
Visibility of different concentrations of EC-ethanol in excised bovine liver. (**a**) Representative B-mode images and corresponding binary masks of EC-ethanol throughout its injection procedure illustrate the real-time depot formation and immediate echogenicity of the gel distribution. Red lines denote needle path before and after injection. Scale bars = 5 cm. (**b**) Dice scores for measuring the similarity between binary masks generated from manual depot segmentation vs. automatic segmentation. *N* = 4.

**Fig. 6:**
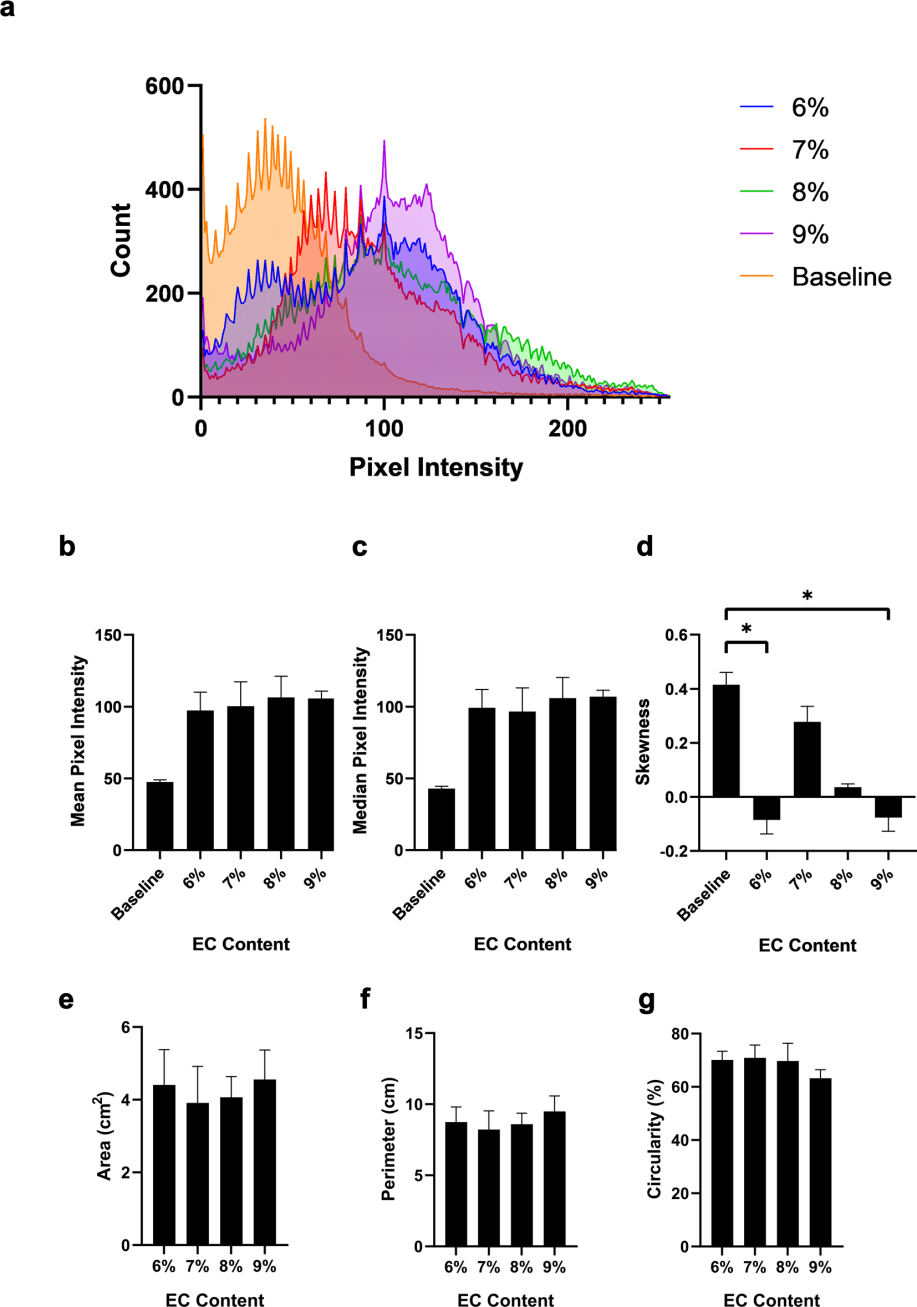
Characterization of the echogenic profile of the EC-ethanol depots in excised bovine liver. (**a**) Line histogram plot depicting the non-zero cumulative pixel intensities observed in the EC-ethanol depot region. Plots depicting the (**b**) mean, (**c**) median, and (**d**) skewness of the pixel intensity distribution. Additional plots showing the (**e**) area, (**f**) perimeter, and (**g**) circularity of the binary masks. All error bars in this figure are SEM. *N* = 3–4. Kruskal-Wallis non-parametric analysis of variance test was performed. *P < 0.05.

*EC-ethanol depots are discernable in liver tissue under ultrasound guidance.* Representative B-mode images and binary masks of the echogenic profile of EC-ethanol depots in excised bovine liver are shown in Fig. 5. Time lapses of the injection process displayed the real-time deposition of the gel, along with subsequent isolation of the depot from the tissue background (Fig. 5a). The Dice scores largely remained consistent, ranging between 0.76 ± 0.03 (6% EC) and a slightly lower score of 0.66 ± 0.07 (12% EC) (*p* > 0.05) (Fig. 5b). The pixel intensities within the ROI of the final B-mode and baseline images were extracted and plotted in Fig. 6a. Contrary to those of the in vitro EC-ethanol depots, both the mean and median pixel intensities did not significantly change as EC content increased (Fig. 6b, c) from a mean of 97 ± 8 (6% EC) to 106 ± 3 (9% EC) and a median of 99 ± 8 (6% EC) to 107 ± 3 (9% EC) (mean ± S.E.M., *n* = 3–4) (*p* > 0.05). However, there was an increase in mean and median pixel intensity with the 9% EC depot when compared to that of the baseline (mean of 48 ± 2 and median of 43 ± 2) (*p* > 0.05) (Fig. 6b, c). The pixel intensities for 6% and 9% EC-ethanol depots displayed negative skewness (−0.09 ± 0.03 and − 0.08 ± 0.03, respectively), which were significantly different from that of the baseline (0.41 ± 0.04, *p* = 0.03 and 0.04, respectively). Both the baseline and 7% EC depots displayed positive skewness (0.41 ± 0.04 and 0.28 ± 0.03, respectively), while 8% EC-ethanol largely followed a normal distribution with a skewness of 0.04 ± 0.01 (Fig. 6d). For the binary mask analysis, the average area (between 3.91 ± 1.01 for 7% EC and 4.56 ± 0.81 cm^2^ for 9% EC) and perimeter (between 8.22 ± 1.31 for 7% EC and 9.49 ± 1.09 cm for 9% EC) for all EC-ethanol ratios remained consistent across EC concentrations (*p* > 0.05) (Fig. 6e, f), similar to that of the EC-ethanol depots in vitro. In contrast with what was observed in the agar phantoms, however, the circularity of the depots in tissue only decreased slightly as EC concentration increased, going from 70 ± 3% (6% EC) to 63 ± 3% (9% EC) (*p* > 0.05) (Fig. 6g).

**Fig. 7:**
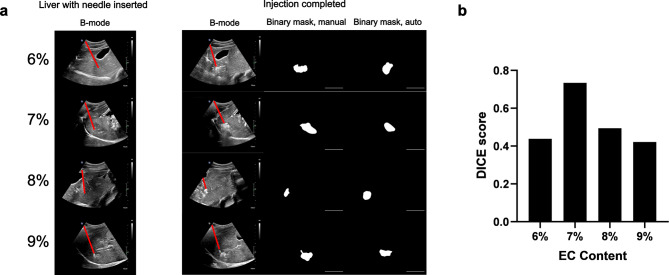
Visibility of EC-ethanol under ultrasound imaging in post-mortem swine liver. (**a**) Representative B-mode images and corresponding binary masks of EC-ethanol after completion of injection. Red lines denote needle path. Scale bars = 5 cm. (**b**) Dice score for measuring the similarity between binary masks generated from manual depot segmentation vs. automatic segmentation. *N* = 1.

**Fig. 8:**
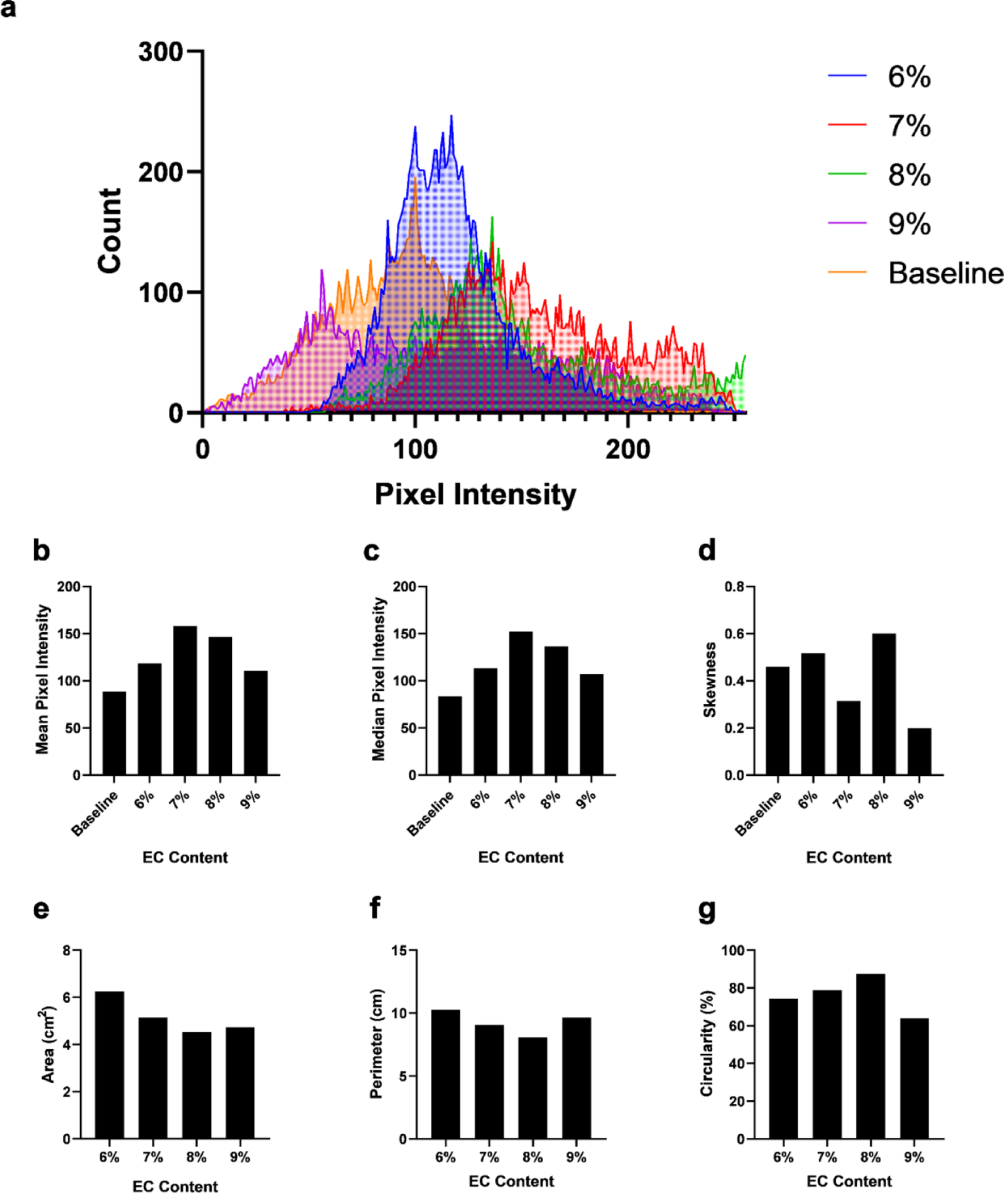
Characterization of the echogenic profile of the EC-ethanol depots percutaneously injected and imaged in swine liver. (**a**) Line histogram plot depicting the non-zero cumulative pixel intensities observed in the EC-ethanol depot region. Plots depicting the (**b**) mean, (**c**) median, and (**d**) skewness of the pixel intensity distribution. Additional plots showing the (**e**) area, (**f**) perimeter, and (**g**) circularity of the binary masks. *N* = 1.

*Percutaneous injections of EC-ethanol into in situ swine liver tissue can be visualized with ultrasound imaging.* The distribution profile of EC-ethanol delivered percutaneously into the liver of an intact pig carcass and the corresponding binary masks for the resulting depots are shown in Fig. 7a. The Dice scores ranged from 0.42 (9% EC) to 0.73 (7% EC) (Fig. 7b). The pixel intensities within the ROI of the final B-mode images were extracted and plotted in Fig. 8a. The mean and median pixel intensities arranged between a mean of 111 (9% EC) to 158 (7% EC) and a median of 107 (9% EC) to 152 (7% EC) (Fig. 8b, c). All EC concentrations generated higher intensities than that of the baseline (84). The pixel intensities of all EC-ethanol depots displayed positive skewness (Fig. 8d), along with a closer shift to a normal distribution compared to baseline (0.46) as EC content was increased from 6% (0.52) to 9% (0.20). For the binary mask analysis, the area ranged between 4.5 and 6.3 cm^2^, while the perimeter ranged between 8.1 and 10.3 cm (Fig. 8e, f). The depots were similar in size to those determined in the excised liver ablation study. All depots maintained a circularity of at least 60% (Fig. 8g), similar to what was observed with the depots in the ex vivo liver study.

## Discussion

Ultrasound-guided ethanol ablation has the potential to be an accessible treatment option for HCC patients in LMICs due to the ubiquity of ethanol in clinics and low procedural cost to perform the treatment. However, the propensity of ethanol to leak from the injection site hampers its ablative efficacy and often requires multiple procedures and injections to effectively ablate the tumor. Additionally, the ethanol liquid is not inherently echogenic^5,23,24^, rendering biodistribution difficult to monitor. To mitigate these problems, a new formulation combining EC with ethanol was developed to rapidly induce gel formation^9^ and enable ultrasound monitoring of the injectate in the presence of tissue. Ultrasound imaging experiments and spatiotemporal analyses within agar-based phantoms, excised liver tissue, and in an intact swine post-mortem carcass, demonstrated that EC-ethanol depot concentrations can be readily tuned for optimal echogenicity within various tissue contexts. The echogenicity of EC-ethanol varied with EC content within the formulation. The gel depot became more echogenic as the % EC increased when injected into agar phantoms. Specifically, a 1.5-fold increase in mean and median pixel intensity within the gel depot was observed as EC content increased from 6 to 12% (Fig. 4b, c). This is corroborated by the significant decrease in mean skewness from 6 to 12% EC as the higher EC-ethanol solutions displayed more normal distributions of the pixel intensities (Fig. 4d). However, at ratios of 8% EC-ethanol and higher, some acoustic artifacts became apparent that obscured parts of the depot in the distal field of the B-mode image (Fig. 3a). These artifacts were also captured by the irregular shapes of the binary masks (Fig. 3c) and decreased in depot circularity as EC content increased beyond 8% (Fig. 4g). This is likely due to the increasingly dense gel network that disrupted and scattered the transmitted sound waves. This caused a mismatch in the acoustic impedance between the gel network and tissue, resulting in subsequent echogenicity and shadowing artifacts. While lower concentrations of EC may avoid the acoustic artifacts (e.g., under 6%), sufficient gelation would not be achieved^9^. Thus, there exists an optimal ratio that achieves both ample gelation and echogenicity within tissue substrates.

When injecting EC-ethanol into liver tissue, the depot characteristics of the gel remained consistent across the 6–9% EC concentrations tested. For the infusion parameters selected, depots for all EC concentrations reached mean areas of approximately 4 cm^2^ (Fig. 6e), including one depot that exceeded 6 cm^2^ (Fig. 8b), which are comparable in size to that of typical HCC tumors suitable for ablation^25^. The depots of all ratios exhibited signs of acoustic shadowing and partial obscuration of the liver tissue background in the far field, though the injected region remained detectable with the segmentation algorithm (Fig. 2b). The pixel intensities within the ROIs for the liver images followed more of a normal distribution, as indicated by the lower magnitude of skewness for injections into liver (Figs. 6d and 8d) compared to injections into a phantom (Fig. 4d), likely due to the heterogeneity of the tissue background microstructures contributing to the overall acoustic profile of the EC-ethanol depots. These results define EC-ethanol’s unique echogenic signature within liver tissue. Future studies could implement different ultrasound contrast modes to dynamically examine EC-ethanol deposition in the liver and further characterize the tissue environment and microstructures.

In terms of detecting EC-ethanol depots in vitro, a benefit of using the agar substrate to examine the EC-ethanol echogenic signal was that it did not generate any significant background signals. This feature allowed for isolating all observed readings and artifacts from the EC-ethanol depot without any confounding variables; the high Dice scores corroborated the similar performance of the automatic segmentation method with that of the manual segmentation (Fig. 3d). On the other hand, for the EC-ethanol depot imaging in liver tissue, the inherent tissue background and presence of the biopsy needle all resulted in multiple echogenic compositions in the images that diversified the acoustic profile of the injections and thus required a temporal-based algorithm to successfully isolate and segment the depot. While the possibility of using a state-of-the-art, zero-shot (i.e., no training is needed) method for depot segmentation was considered^26^, the model’s attempts to identify the depot exhibited low specificity (Supplementary Fig. S2). Moreover, the model required manual prompting (i.e., manually drawing a region-of-interest) to produce accurate segmentation results, instead of a semi-automatic procedure. The Dice scores were relatively lower than those calculated for the in vitro images (Fig. 5b), but scores were still above 0.7, which was considered adequate for ultrasound images where tissue background may interfere with ROI detection^27,28^. For the percutaneous injections, only the 7% EC injection achieved a Dice score higher than 0.7 when comparing automatic and manual segmentation techniques (Fig. 7b), with the other injections falling closer to 0.4 (Fig. 7b). This may be due to the fact that the background noise is greater than that of both the agar and excised liver studies, as evidenced by the increased mean and median pixel intensities (Fig. 8b, c) and subsequent heightened difficulty to detect and segment the depot. Future studies will optimize the image processing algorithm to recognize the biopsy needle tip to predetermine the expected location of the EC-ethanol injectate and define a smaller ROI and be less susceptible to artifacts. Furthermore, the algorithm can be re-implemented using a compiler-based language such as C/C + + or leveraging multi-threading for faster execution. There is also currently ongoing work to train a convolutional neural network for ultrasound image feature interpretation, which may allow for streamlined image acquisition and segmentation analyses.

The successful execution of the percutaneous injection of EC-ethanol into the liver of the intact swine carcass established feasibility for visualization in situ. The operator was able to sweep the swine abdomen and locate suitable locations throughout the liver to inject EC-ethanol and image via ultrasound. One imaging challenge was keeping the needle, with the tip in the injection site, within the transducer imaging plane throughout the full duration of the injection to obtain spatiotemporal consistency of the obtained B-mode images for image analysis. Employing a needle guide or robotic arm to fix the needle or transducer position may mitigate this issue in subsequent studies. This study sets the foundation for performing EC-ethanol injections in a large animal model in vivo, in anticipation of translation to human treatment of HCC. EC-ethanol has the advantage of using low-cost consumables and requires little maintenance to perform the ablation. This study established a procedural workflow to perform EC-ethanol injections under ultrasound guidance and an automated segmentation algorithm to characterize the injection depot. The process is cost-effective and requires minimal equipment, suggesting suitability of this tumor ablation modality for translation into clinics situated in LMICs.

There were several limitations associated with this study. First, only 30-second time lapses of the liver injections were captured, and all temporal information in between were not preserved. Future studies will obtain continuous video footage of the entire injection process to assist the algorithm in isolating the depot and filtering out tissue background to improve depot segmentation. Second, the features selected to examine the EC-ethanol depots served to establish the morphometric and echogenic properties of EC-ethanol in liver tissue and set the groundwork for translating EC-ethanol treatment into a clinical setting. In the future, we will explore other features in larger data sets, including the shape or bimodal aspects observed in the pixel intensity histograms. Third, it was not possible to accurately compare EC-ethanol depot size and morphometry in gross pathologic liver specimens and tissue sections to measurements using ultrasound imaging, due to leakage or smearing of the injected EC-ethanol during physical manipulation. In the future, measurements of EC-ethanol depots in liver may be compared to corresponding volumetric measurements acquired using transducer manipulation techniques or clinical imaging modalities such as CT and MR imaging. Fourth, some echogenic and morphometric properties of EC-ethanol injections may be influenced by biophysical properties that may differ between healthy tissues used in this study and tumors. Additionally, the feasibility of percutaneous injection was demonstrated on one swine post-mortem. In larger future studies, we will percutaneously inject and image EC-ethanol depot formation in animal HCC models in vivo to further assess EC-ethanol depot imageability within the context of HCC. Finally, EC is known to spontaneously degrade within a few weeks up to 6 months, as shown previously in clinical studies when used as a sclerotherapy to treat venous malformations^29,30^. However, there is little to no information on the retention time and clearance of EC from liver tissue after EC-ethanol ablation. Thus, future studies will investigate EC-ethanol long-term degradation in vivo.

## Conclusion

Injecting EC-ethanol in tissue generated an echogenic depot when visualized under ultrasound imaging. While the signal strength of the depot is closely tied to EC content within the injectate in vitro, the overall pixel intensities for 9% EC-ethanol depots and above plateaued and produced acoustic shadowing artifacts that drastically lowered depot and tissue visibility. In liver tissue, EC-ethanol injections and depot formation could be monitored in real-time to assess clinically relevant depot size and localization within tissue. Additionally, EC-ethanol could be delivered to the liver percutaneously and be readily visualized using ultrasound imaging during depot formation. Semi-automatic segmentation of EC-ethanol injections on ultrasound imaging, described herein, could potentially enable assessment of tumor coverage and estimation of treatment adequacy of EC-ethanol ablation. Furthermore, future studies will focus on the optimization and real-time deployment of the segmentation algorithms, including the implementation of learning-based methods (e.g., neural network) and superior computation speed enabled with GPU acceleration. This study establishes the groundwork for future work optimizing EC-ethanol delivery and acoustic imageability in vivo, with eventual translation to patients worldwide.

## Electronic supplementary material

Below is the link to the electronic supplementary material.

Supplementary Material 1

## Data Availability

The authors confirm that the data supporting the findings of this study are available within the article. For additional supporting materials, please contact the corresponding author.
