# Supplementary material for: Evaluating the echogenicity of ethyl cellulose-ethanol gel for tracking biodistribution during liver ablation
Source: Sci Rep. 2025 Jul 15;15:25596. doi: 10.1038/s41598-025-11336-9 (PMC12264171; doi:10.1038/s41598-025-11336-9)
Supplement: Supplementary file 1 — Supplementary Material 1 [file 41598_2025_11336_MOESM1_ESM.docx]

Supplementary Figure S1


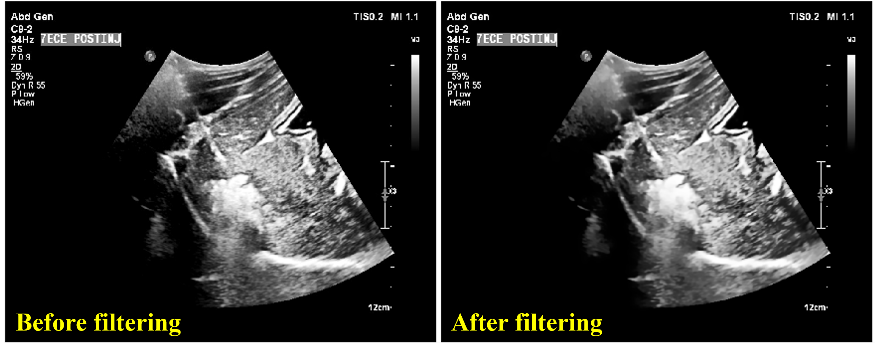


**Supplementary Figure S1.** Effect of non-local means filter when applied on a representative percutaneous swine liver image.

Supplementary Figure S2


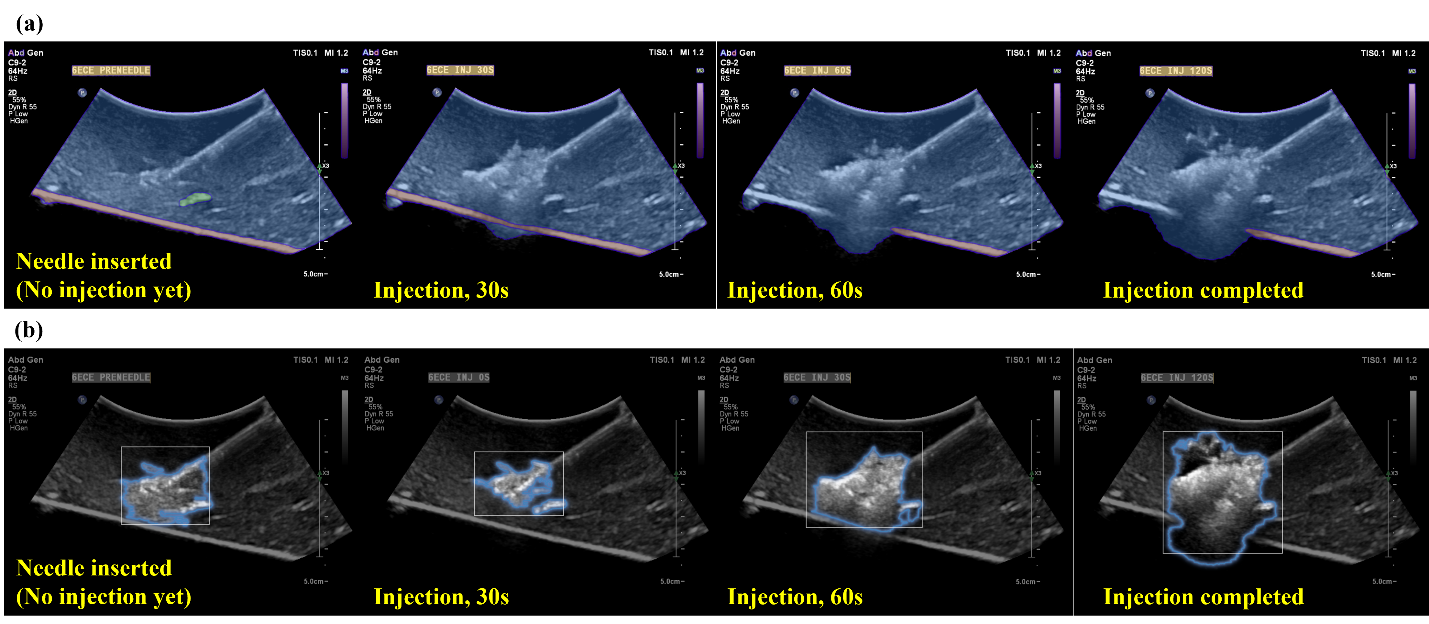


**Supplementary Figure S2**. Testing pretrained foundational model for depot segmentation on representative bovine liver images. (a) Segmentation without prompting. The blue shaded areas represent the segmented regions which do not correspond to the depot area at all. (b) Segmentation with manual prompting.
